# Circulating Levels of L1-cell Adhesion Molecule as a Serum Biomarker for Early Detection of Gastric Cancer and Esophagogastric Junction Adenocarcinoma

**DOI:** 10.7150/jca.41100

**Published:** 2020-07-11

**Authors:** Ling-Yu Chu, Yu-Hui Peng, Tian Yang, Wang-Kai Fang, Chao-Qun Hong, Li-Sheng Huang, Li-Yan Xu, En-Min Li, Yi-Wei Xu, Jian-Jun Xie

**Affiliations:** 1 Department of Biochemistry and Molecular Biology, Shantou University Medical College, Shantou, China.; 2 Department of Clinical Laboratory Medicine, the Cancer Hospital of Shantou University Medical College, Shantou, China.; 3 Department of Gastrointestinal Surgery, the First Affiliated Hospital of Shantou University Medical College, Shantou, China.; 4 Department of Oncological Laboratory Research, the Cancer Hospital of Shantou University Medical College, Shantou, China.; 5 Department of Radiation Oncology, the Cancer Hospital of Shantou University Medical College, Shantou, China.; 6 Institute of Oncologic Pathology, Shantou University Medical College, Shantou, China.

**Keywords:** gastric cancer, esophagogastric junction cancer, L1CAM, serum biomarker, early diagnosis

## Abstract

**Background:** Low serum L1 cell adhesion molecule (L1CAM) has been found in several malignant tumors. Here, we aimed to evaluate the diagnostic potential for serum L1CAM in patients with gastric cancers (GC) and esophagogastric junction adenocarcinoma (EJA).

**Methods:** Enzyme-linked immunosorbent assay (ELISA) was carried out to detect L1CAM level in sera of 148 GC patients, 59 EJA patients and 148 healthy controls. Receiver operating characteristics (ROC) was employed to evaluate diagnostic accuracy.

**Results:** The concentrations of serum L1CAM were significantly lower in GC and EJA than those in healthy controls (P<0.001). Detection of L1CAM provided a sensitivity of 83.1%, a specificity of 62.2%, and an area under the curve (AUC) of 0.769 (95% CI: 0.715-0.823) in diagnosing GC, and a sensitivity of 66.1%, a specificity of 62.2%, and an AUC of 0.672 (95% CI: 0.590-0.755) in diagnosing EJA. Similar results were observed in the diagnosis of early-stage GC (0.681 (95%CI: 0.596-0.766)) and early-stage EJA (0.674 (95%CI: 0.528-0.820)). Analysis of clinical data showed that the levels of L1CAM were significantly associated with lymph node metastasis in GC (P<0.05).

**Conclusions:** Our study showed that serum L1CAM might be a diagnostic biomarker for GC and EJA.

## Introduction

Gastric cancers and esophagogastric junction cancers are common digestive tract malignancies all over the world. Gastric cancer is the fifth leading incident cause of cancer and the third leading cause of cancer-related death worldwide. It was estimated that 1,033,701 new cases were diagnosed as gastric cancer and 782,685 people cases died of it in 2018 1. Despite a worldwide decline in the incidence of gastric cancer, incidence of China remains high 1-3. On the other hand, in the worldwide, the incidence of esophagogastric junctional cancer has increased rapidly in recent decades 1,3. Some studies have shown that the incidence of esophagogastric junctional cancer in China is higher than that in Western countries 1,3,4. The vast majority of gastric cancers and esophagogastric junctional cancers are adenocarcinomas 5,6. Despite the use of multimodal treatments such as radical surgery, chemotherapy and radiotherapy, gastric cancers (GC) and esophagogastric junction adenocarcinomas (EJA) patients still demonstrate extremely poor survival rate 7-9. Due to lack of screening strategies for timely diagnosis, many patients are diagnosed with advanced GC and EJA 7-10. Therefore, early detection of GC and EJA is the key to improve survival rate, and requires an effective screening program.

Currently, the diagnosis of digestive tract tumor relies on endoscopy of symptomatic patients 11. At present, the diagnostic rate of early-stage GC in China is less than 10.0%, which has gradually increased from recent years, but it is still far lower than the detection rate of 70.0% in Japan 12. As endoscopic diagnosis is invasive, very few people are willing to do this examination 13,14. Because the lesions of early-stage GC and EJA are usually relatively small and the changes under endoscopy are relatively subtle, it is difficult to diagnose early-stage GC and EJA by endoscopy. Serological tests are generally considered to be the simplest and the most non-invasive methods for the advantages of allowing high-throughput screening and sample collection. It has been widely developed in clinical diagnosis. However, many of the well-known serum cancer-associated biomarkers, such as carcinoembryonic antigen (CEA), cancer antigen 125 (CA125), cancer antigen 242 (CA242) and cancer antigen 19-9 (CA19-9), are not sensitive and specific enough for screening GC and EJA 15,16. Therefore, we would like to find a better tumor biomarker in this study for early detection of GC and EJA.

L1 cell adhesion molecule (L1CAM), also designated as CD171, is a 200-220-kDa type 1 membrane glycoprotein from immunoglobulin superfamily, known for its roles in nerve cell functions 17,18. L1CAM protein is composed of 1256 amino acids, including extracellular domain, transmembrane domain and intracellular domain. The extracellular portion, consisting of six immunoglobulin domains and five fibronectin repeats (type III), is connected to a small intracellular domain by a transmembrane helix 17,19-21. In recent years, it has been reported that L1CAM is overexpressed in various types of cancer cells and acts as a driving factor of carcinogenesis 22. Additionally, L1CAM has been found to be a potential biomarker in several types of human cancers 21,23, including esophageal squamous cell cancer (ESCC) 22, gastrointestinal stromal tumors (GIST) 24, uterine and ovarian cancers 25 and other less common types of cancer. For example, high soluble L1 levels predict poor prognosis of GIST, and may thus be a promising tumor marker that can help to individualized therapy in GIST 24. Fogel et al. 25 measured the serum L1CAM concentration by ELISA and found it was significantly up-regulated in the serum of ovarian and uterine carcinomas patients compared to the control group. Studies showed that the L1CAM could be used as a biomarker for ovarian and uterine carcinomas associated with poor clinical outcome. However, the diagnostic value of serum L1CAM for GC and EJA has rarely been reported. Therefore, we tried to explore the relationship between serum L1CAM and GC and EJA.

## Materials and Methods

### Study samples

In this study, 148 serum samples of GC patients were collected from the Cancer Hospital of Shantou University Medical College, from June 2012 to November 2016. 59 serum samples of EJA patients were collected from the First Affiliated Hospital of Shantou University Medical College, from January 2018 to November 2018. 148 normal controls were selected from the biobank of Shantou University Medical College, who were healthy subjects with no previous malignant disease enrolled in the Cancer Hospital of Shantou University Medical College and the First Affiliated Hospital of Shantou University Medical College. All the GC and EJA serum samples were collected immediately before any tumor-related treatment and the healthy controls were eligible blood donors with no evidence of cancers. The serum samples were allowed to coagulate at room temperature for 30 mins before centrifuged at 1,250 *g* for 5 minutes. Then they were stored at a temperature of -80 °C until the experiment started.

GC and EJA were diagnosed on the basis of computed tomography or gastroscopy. Tumor stage was defined according to the eighth edition of the American Joint Committee on Cancer (AJCC) Cancer Staging Manual 26. In the study, we classified tumors with AJCC stage 0 + Ⅰ + Ⅱ as early-stage GC and EJA. The present work was approved by the Ethics Committee of the Cancer Hospital of Shantou University Medical College and the Ethics Committee of the First Affiliated Hospital of Shantou University Medical College and informed to all participants were obtained during blood collection. All work was complied with the principles of the Helsinki Declaration.

### Enzyme-Linked Immunosorbent Assay (ELISA) for L1CAM

The levels of Serum L1CAM were detected by ELISA Kit (Sino Biological Inc, cat.no. SEK10140, Beijing, USA). We prepared reagents, samples and standard products according to the manufacturer's instructions. Briefly, 96-well ELISA plates (Biohaotian, cat. no. HT081, Jiangsu, China) were coated with 100 µl diluted capture antibody (2 µg/ml) and incubated overnight at 4 °C. The plates were washed by microplate washer (Thermo Fisher Scientific), and then blocked using 300 µl of blocking buffer and incubated at room temperature for 1 hour. After washing, 100 µl of serum samples (a 200-fold dilution) and standards were added in per well and incubated for 2 hours at room temperature. The concentrations of the L1CAM standard curve were 0 pg/ml, 47 pg/ml, 94 pg/ml, 188 pg/ml, 375 pg/ml, 750 pg/ml, 1500 pg/ml, and 3000 pg/ml, respectively. After removing the liquid and washing, 100 µl of detection antibody (0.5 µg/ml) was added in per well and incubated for 1 hour at room temperature. Next, after washing, 200 l substrate solution was added to each well and then incubated at room temperature for 20 minutes. Color formation was stopped by stop solution, and the optical density (OD) value was read at wavelength of 450 nm and 590 nm on a plate microplate reader (Thermo Fisher Scientific). Use the standard curve method to convert the OD value (Supplementary Table S1) to the concentration and multiply it by the dilution factor. Each serum sample was tested in duplicate and averaged for analysis.

### Statistical Analysis

Data analyses were performed using SPSS (version 19.0), GraphPad Prism 7.0 software and Microsoft Excel. The concentrations of serum L1CAM were obtained with a standard curve. A nonparametric Mann-Whitney's U test was used to compare the difference of serum levels of L1CAM between each group pair. Results were expressed as mean ± standard deviation. Receiver Operating Characteristic (ROC) curves were plotted and area under the ROC curve (AUC) with 95% confidence interval (CI) was calculated to analyze the accuracy of diagnostic value. The optimum cut-off values were obtained from the Youden's indexes of the ROC curves, which yield maximum values of sensitivity plus (100% - specificity) 27. By using these optimal cutoff values, sensitivity, specificity, positive predictive values (PPV), negative predictive values (NPV), false positive rate (FPR), false negative rate (FNR), positive likelihood ratio (PLR), and negative likelihood ratio (NLR) were calculated. Correlation between clinical parameters and positive rates of serum L1CAM was evaluated with Chi-square test. For all analyses, *P* <0.05 (two-tailed) was considered to be statistically significant.

## Results

### The level of serum LICAM in GC, EJA patients and healthy controls

**Figure 1:**
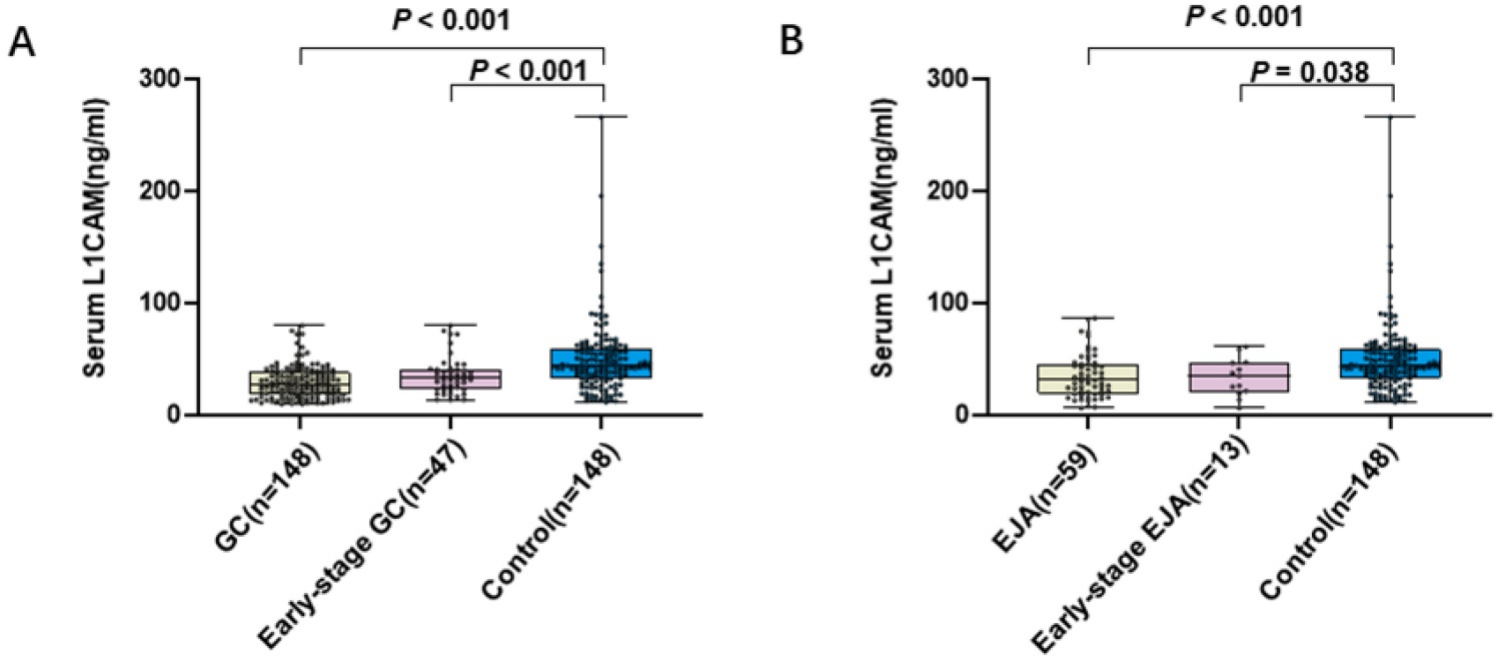
Level of serum L1CAM. **A.** Scatter plot and box plot of OD values of L1CAM from GC serum and normal serum. **B.** Scatter plot and box plot of OD values of L1CAM from EJA serum and normal serum. Black horizontal lines are means.

To evaluate the diagnostic potential of L1CAM, 355 serum samples were tested, including GC patients (n = 148), EJA patients (n = 59) and healthy control subjects (n = 148). The mean age of GC patients, EJA patients and healthy controls in our present study were 58 years, 63 years and 58 years, respectively (Table 1). The levels of L1CAM (mean ± SD) were 28.687 ± 14.162 ng/mL, 34.506 ± 19.408 ng/mL, 35.265 ± 16.300 ng/mL, 33.522 ± 16.972 ng/mL and 49.325 ± 31.722 ng/mL in GC, EJA, early-stage GC, early-stage EJA and healthy control, respectively (Table 2). Compared with normal controls, we noted that L1CAM levels were statistically significantly lower in GC patients, early-stage GC than those in controls (Figure 1A). Similar results were observed in EJA (Figure 1B). However, there was no difference in serum L1CAM between GC and EJA (Supplementary Figure S1).

### Diagnostic capacity of L1CAM in all GC and all EJA

**Figure 2:**
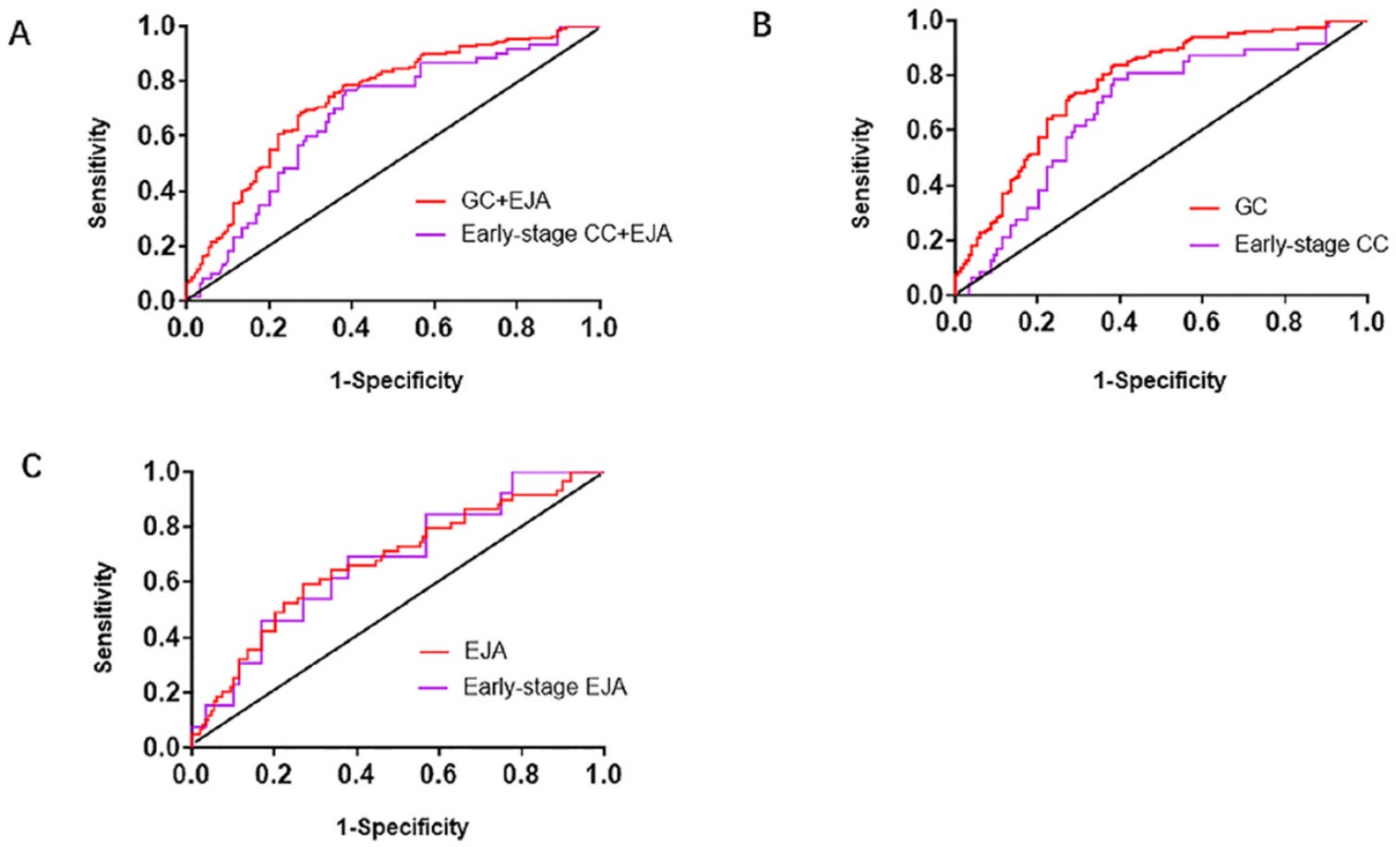
Receiver operating characteristic (ROC) curve analysis in the diagnosis of GC+EJA, GC, EJA, early-stage GC+EJA, early-stage GC and early-stage EJA. **A.** The ROC curves for serum L1CAM in patients with GC+EJA and early-stage GC+EJA compared with those in normal control group. **B.** The ROC curves for serum L1CAM in patients with GC and early-stage GC compared with those in normal control group.** C.** The ROC curves for serum L1CAM in patients with EJA and early-stage EJA compared with those in normal control group. The area under the block line is 0.5, for reference.

We generated ROC curves to assess diagnostic capacity of serum L1CAM in GC and EJA. According to the ROC curve, the optimized cutoff value for GC and EJA was 40.720ng/ml. When combined with GC and EJA, we acquired an AUC of 0.742 (95%CI: 0.689-0.794) with a sensitivity/specificity of 78.3% (95%CI: 71.9%-83.6%) / 62.2% (95%CI: 53.8%-69.9%). In the early stage, the AUC for L1CAM diagnosis of GC and EJA was 0.679 (95% CI: 0.601-0.758) and the sensitivity / specificity was 75.0% (95% CI: 61.9% -84.9%) / 62.2% (95% CI: 53.8 % -69.9%) (Figure 2A and Table 3). When we analyzed the diagnostic values of serum LICAM in GC and EJA separately, the AUC for GC was 0.769 (95%CI: 0.715-0.823), and 0.672 (95%CI: 0.590-0.755) for EJA. In the early stage, the AUCs of 0.681 (95%CI: 0.596-0.766) and 0.674 (95%CI: 0.528-0.820) were obtained for GC and EJA, respectively (Figure 2 and Table 3). Using a cutoff value of 40.720ng/ml, the L1CAM has sensitivities of 83.1%, 66.1%, 76.6% and 69.2%, has specificities of 62.2%, 62.2%, 62.2% and 62.2% in the GC, EJA, early-stage GC and early-stage EJA patients, respectively (Table 3). For better interpretation on clinical value, we performed predictive values and likelihood ratios for L1CAM in the GC and EJA diagnosis, as shown in Table 3.

### Correlation between serum concentration of L1CAM and clinicopathological features

The relationships of the levels of serum L1CAM and clinicopathological features were showed in Tables 4 and 5. The levels of L1CAM in GC were significantly associated with lymph node metastasis (*P*<0.05), but not with other clinical data, including age, gender, smoking, depth of invasion, metastasis and TNM stage (Table 4). However, the levels of L1CAM had no statistical correlation with clinical data of EJA (Table 5). We also used the mean method to analyze the relationship between serum L1CAM concentration and clinicopathological characteristics. The results are shown in supplementary tables S2 and S3.

## Discussion

In recent years, the morbidity and mortality of GC and EJA has ranked among the top in China 2-4. Helicobacter pylori infection, genetic and environmental factors are high risk factors for GC 28,29. Gastroesophageal refluxes disease (GERD), as well as increasing body weight and obesity, were strongly associated with an increased risk of EJA 4, but associations between H pylori and EJA are unclear 4,29. The development of GC includes inflammation, gland atrophy, intestinal metaplasia and dysplasia, and it is considered that the carcinogenic process of EJA is very similar to that of GC patients 4,30. Relevant statistical results showed that the 5-year survival rate of domestic GC patients after surgery was 30.0-50.0%. If early diagnosis can be made, the cancer cells are still confined to the gastric mucosa and submucosa, and the 5-year survival rate can reach to over 90.0% 10,15. According to the US Cancer Registry, EJA's 5-year survival rate is generally 10.0-15.0%. In early EJA patients, the 5-year survival rate can increase to 25.0 -30.0% 9. Thus, early diagnosis and early treatment are the key to improving the survival rate of GC and EJA. In recent years, with the in-depth study of clinical medicine, the detection of serum tumor markers has been widely developed in clinical diagnosis. Tumor markers are a class of substances that reflect the presence of tumors. When these substances reach a certain level, they can reveal the existence of certain tumors 15,16,31. This feature makes early diagnosis of GC and EJA possible.

Cell adhesion molecules (CAMs) are cell surface proteins, and they mediate cell-to-cell and/or cell-to- extracellular matrix (ECM) interactions 18. The expression of L1CAM can promote the remodeling of extracellular matrix and enhance the chemotaxis of tumor cells to the extracellular matrix, both of which promote tumor invasion and metastasis 18-20,23. At present, L1CAM protein has been studied in evaluating the malignancy and prognosis of ovarian, endometrial and melanoma tumors 18,25,32,33. The expression of L1CAM protein in cancer tissues and adjacent normal tissues of patients with breast and colorectal cancer was analyzed by immunohistochemical method. The results showed that the expression of L1CAM in cancer tissues was significantly higher than that in adjacent normal tissues 34,35. These results suggest that L1CAM is involved in the development of tumors. Studies have shown that L1CAM is highly expressed in certain tumor tissues, but not in all tumor tissues, and is limited to certain tumors or subtypes of certain tumors, such as L1CAM is high expression in gastrointestinal stromal tumors (GIST) and uterine and ovarian cancers, but low expression in esophageal squamous cell carcinoma (ESCC) 22,24,25. In our study, the concentrations of serum L1CAM were significantly lower in GC and EJA than those in healthy controls (*P*<0.001). We speculate that this difference may be due to different expression patterns and different histopathological types of L1CAM in different types of cancer 22. This has certain significance for differential diagnosis of different tumors. Therefore, L1CAM has broad application prospects as a diagnostic marker for tumors.

This study demonstrated the potential role of serum L1CAM in the early detection of GC and EJA. Serum L1CAM performed a diagnostic value in GC with AUC of 0.769, sensitivity of 83.1% and specificity of 62.2%. As for the EJA, serum L1CAM expression levels demonstrated AUC values of 0.672, associated with 66.1% sensitivity and 62.2% specificity. Similar results were observed in the early-stage GC and early-stage EJA. Combined with GC and EJA, the diagnostic value of AUC was 0.742, the sensitivity was 78.3% and the specificity was 62.2%. In conclusion, the diagnostic value of serum L1CAM on GC seems better than that of EJA, which may be due to the fact that the sample size of GC is larger than that of EJA. If the sample size of EJA is increased, the diagnostic value may be improved. In our previous study, serum L1CAM levels were measured by ELISA in 94 normal and 191 ESCC patients, and the results showed that it achieved the AUC of 0.644 for ESCC and 0.629 for early-stage ESCC 22. These findings suggest that serum L1CAM might be a marker for the diagnosis of gastrointestinal cancer.

Although L1CAM has been shown to be a useful biomarker in the diagnosis of early GC and EJA, its relatively low specificity may limit its clinical application in screening patients with asymptomatic early GC and EJA. However, single tests of serum L1CAM couldn't meet the requirement of clinical practice. Studies have shown that combined detection of multiple serum proteins would increase the sensitivity or specificity in gastrointestinal tract malignancies compared with a single biomarker. As reported, the combination of cancer antigen (CA724), CEA and CA19-9 can improve the diagnosis of GC, increasing the sensitivity from 47.0% to 74.0%. These are commonly used as serum tumor markers in GC 15,36. The biomarker CA242 also has a high sensitivity up to 44.0 % in GC 37. Therefore, one of the limitations of our study is that L1CAM is not used in combination with these common markers to diagnose GC and EJA. Moreover, highly sensitive L1CAM can be used for early diagnosis of GC and EJA, thereby improving the prognosis of GC and EJA. Because the specificity of L1CM is relatively low, we hope that L1CAM could be used as a potential biomarker to combine with some established tumor markers (such as CEA, CA724, CA19-9, CA242) for the diagnosis of GC and EJA. At present, the examination of cancer of the digestive tract mainly depends on endoscopy. If the method of this experiment is combined with endoscopy, the common diagnosis might help to improve the diagnosis rate of early-stage GC and EJA. As for the relationship between serum L1CAM and the clinical data of GC and EJA, we found that the level of serum L1CAM was associated to lymph node metastasis (*P*<0.05) in GC but there was no statistical difference with EJA. These results suggest that L1CAM may play an important role in lymph node metastasis. It is possible that L1CAM, as a member of cell adhesion molecule family, can promote cancer cell trans endothelial metastasis through receptor-ligand interaction 38. As the age of cancer cases and healthy controls were not well matched, further study could be conducted with corresponding age. However, our study is single and the sample size is small, which may lead to bias. We believe that the power of our study on evaluating serum L1CAM for GC and EJA will be significantly improved if large sample studies are conducted in multiple institutions.

## Conclusion

In summary, our study provided useful information for the diagnostic value of serum L1CAM in GC or EJA, and demonstrated that serum L1CAM might serve as a potential biomarker for early detection of GC and EJA.

## Supplementary Material

Supplementary figure and tables.

## Figures and Tables

**Table 1:** Participant information and clinicopathological characteristics

Group	GC patients (n=148)	P*	EJA patients (n=59)	P*	Normal controls (n=148)
**Age (years)**					
Mean ± SD	58 ±11	0.899	63 ±10	0.02	58 ±11
Range	26-81		22-81		29-84
**Gender**					
Male	99	0.714	45	0.113	96
Female	49		14		52
**Smoke**					
Yes	66		13		
No	82		42		
Unknown	0		4		
**Depth of tumor invasion**					
Tis	0		2		
T1	14		3		
T2	5		2		
T3	28		2		
T4	94		40		
Unknown	7		10		
**Lymph node metastasis**					
N0	43		14		
N1	25		8		
N2	41		7		
N3	31		20		
Unknown	8		10		
**Distant metastasis**					
Yes	19		14		
No	123		35		
Unknown	6		10		
**TNM stage**					
0	0		2		
Ⅰ	15		5		
Ⅱ	31		6		
Ⅲ	68		21		
Ⅳ	31		15		
Unknown	3		10		

*Compared with normal controls. GC: Gastric cancers; EJA: Esophagogastric junction adenocarcinomas.

**Table 2:** Comparison between five groups

	N	Serum L1CAM expression	*P* value*
		Mean ± SD	
GC	148	28.687 ±14.162	<0.001
Early-stage GC (0+I+II)	47	35.265 ±16.300	<0.001
Advanced-stage GC (III+IV)	101	25.625 ±11.951	<0.001
EJA	59	34.506 ±19.408	<0.001
Early-stage EJA (0+I+II)	13	33.522 ±16.972	0.038
Advanced-stage EJA (III+IV)	46	34.784 ±20.207	<0.001
Normal controls	148	49.325 ±31.722	

*Compared with normal controls. GC: Gastric cancers; EJA: Esophagogastric junction adenocarcinomas.

**Table 3:** Evaluation of the detection value of L1CAM in the diagnosis of GC and EJA

Group	AUC (95%CI)	Sensitivity	Specificity	FPR	FNR	PPV	NPV	PLR	NLR
**All-stage**									
GC vs. NC	0.769 (0.715-0.823)	83.1%	62.2%	37.8%	16.9%	68.7%	78.6%	2.20	0.27
EJA vs. NC	0.672 (0.590-0.755)	66.1%	62.2%	37.8%	33.9%	41.1%	82.1%	1.75	0.55
GC+EJA vs. NC	0.742 (0.689-0.794)	78.3%	64.2%	35.8%	21.7%	74.3%	67.2%	2.07	0.35
**Early-stage**									
GC vs. NC	0.681 (0.596-0.766)	76.6%	62.2%	37.8%	23.4%	39.1%	89.3%	2.02	0.38
EJA vs. NC	0.674 (0.528-0.820)	69.2%	62.2%	37.8%	30.8%	13.8%	95.8%	1.83	0.49
GC+EJA vs. NC	0.679 (0.601-0.758)	75.0%	62.2%	37.8%	25.0%	44.6%	86.0%	1.98	0.40

AUC, area under the curve; 95% CI, 95% confidence interval; GC: gastric cancer; EJA: esophagogastric junctional adenocarcinomas; NC, normal controls; FNR, false negative rate; FPR, false positive rate; PPV, positive predictive value; NPV, negative predictive value; PLR, positive likelihood ratio; NLR, negative likelihood ratio.

**Table 4:** Association of L1CAM level with GC patients' clinicopathologic features

Variables	L1CAM protein			
	All cases	Low level	High level	*P*
**Age**				
>58	82	64	18	0.067
≤58	66	59	7	
**Gender**				
Male	99	80	19	0.288
Female	49	43	6	
**Smoke**				
Yes	66	51	15	0.089
No	82	72	10	
**Alcohol**				
Yes	27	21	6	0.414
No	121	102	19	
**Size of tumor**				
>5cm	41	37	4	0.33
≤5cm	60	49	11	
Unknown	47	37	10	
**Depth of tumor invasion**				
T1+T2	19	16	3	0.171
T3+T4	122	103	19	
Unknown	7	4	3	
**Lymph node metastasis**				
N0	43	33	10	0.036
N1	25	18	7	
N2	41	35	6	
N3	31	31	0	
Unknown	8	6	2	
**Distant metastasis**				
Yes	19	16	3	0.99
No	123	102	21	
Unknown	6	5	1	
**TNM stage**				
Early stage (I+II)	46	35	11	0.252
Advanced stage (III+IV)	99	85	14	
Unknown	3	3	0	

Comparisons between groups were conducted with the use of person's Chi² tests. GC: gastric cancer.

**Table 5:** Association of L1CAM level with EJA patients' clinicopathologic features

Variables	L1CAM protein			
	All cases	Low level	High level	*P*
**Age**				
>63	44	31	13	0.226
≤63	15	8	7	
**Gender**				
Male	45	28	17	0.259
Female	14	11	3	
**Smoke**				
Yes	13	7	6	0.231
No	42	28	14	
Unknown	4	4	0	
**Diabetes**				
Yes	3	1	2	0.174
No	52	34	18	
Unknown	4	4	0	
**Adjuvant therapy**				
Yes	23	15	8	0.962
No	25	17	8	
Unknown	11	7	4	
**Depth of tumor invasion**				
Tis	2	2	0	0.454
T1+T2	5	2	3	
T3+T4	42	28	14	
Unknown	10	7	3	
**Lymph node metastasis**				
N0	14	10	4	0.95
N1	8	5	3	
N2	7	5	2	
N3	20	12	8	
Unknown	10	7	3	
**Distant metastasis**				
Yes	14	9	5	0.956
No	35	23	12	
Unknown	10	7	3	
**TNM stage**				
Early stage (0+I+II)	13	9	4	0.903
Advanced stage (III+IV)	36	23	13	
Unknown	10	7	3	

Comparisons between groups were conducted with the use of person's Chi² tests. EJA: esophagogastric junction adenocarcinomas.

## Abbreviations

GC: gastric cancers
EJA: esophagogastric junction adenocarcinomas
CEA: carcinoembryonic antigen
CA125: cancer antigen 125
CA242: cancer antigen 242
CA742: cancer antigen 742
CA19-9: cancer antigen 19-9
L1CAM: L1 cell adhesion molecule
ESCC: esophageal squamous cell cancer
GIST: gastrointestinal stromal tumors
AJCC: American Joint Committee on Cancer
ELISA: Enzyme-Linked Immunosorbent Assay
OD: optical density
ROC curve: receiver operating characteristic curve
AUC: area under the ROC curve
CI: confidence interval
FPR: false positive rate
FNR: false negative rate
PPV: positive predictive value
NPV: negative predictive value
PLR: positive likelihood ratio
NLR: negative likelihood ratio
GERD: gastroesophageal refluxes disease
CAMs: cell adhesion molecules
ECM: extracellular matrix
