# Supplementary material for: Circulating Levels of L1-cell Adhesion Molecule as a Serum Biomarker for Early Detection of Gastric Cancer and Esophagogastric Junction Adenocarcinoma
Source: J Cancer. 2020 Jul 11;11(18):5395–402. doi: 10.7150/jca.41100 (PMC7391208; doi:10.7150/jca.41100)
Supplement: Supplementary file 1 — Supplementary figure and tables. [file jcav11p5395s1.pdf]

Supplemental material

Circulating Levels Of L1-cell Adhesion Molecule As A Serum Biomarker For Early Detection Of Gastric Cancer And Esophagogastric Junction Adenocarcinoma

Supplementary Figure S1

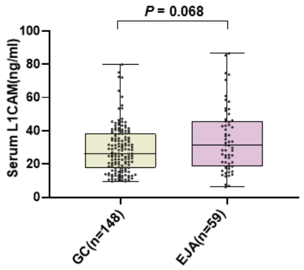

Figure S1

Supplementary Tables(S1,S2,S3)

Table S1. OD value of each sample and its corresponding concentration.

GC number	OD	Expression	EJA number	OD	Expression	NC number	OD	Expression
G2	0.166	22.53312	1	0.239	58.60104	First affiliated hospital of shantou		
G3	0.104	12.8542	2	0.11	28.2524	university medical collgee		
G16	0.14	18.40138	3	0.085	21.03894	2	0.2035	29.77
G17	0.089	10.61008	4	0.094	23.7346	6	0.5645	89.61
G20	0.108	13.45972	5	0.099	25.18016	14	0.1905	27.78
G21	0.224	32.08452	6	0.313	73.79778	26	0.282	42.01
3G001	0.119	15.13936	8	0.063	13.721	27	0.387	59.04
3G002	0.215	30.5738	9	0.095	24.02644	34	0.221	32.46
3G003	0.105	13.0053	10	0.168	42.8258	37	0.304	45.51
3G005	0.092	11.05542	11	0.085	21.03894	38	0.167	24.22
3G006	0.089	10.61008	12	0.169	43.0602	40	0.091	12.98
3G007	0.105	13.0053	13	0.073	17.207	43	0.122	17.51
3G014	0.168	22.85496	14	0.099	25.18016	44	0.25	36.97
3G016	0.203	28.57548	16	0.068	15.5049	45	0.461	71.51
3G018	0.144	19.03054	17	0.1	25.46534	50	0.168	24.37
3G022	0.136	17.77466	18	0.25	60.92274	54	0.603	96.55
3G023	0.114	14.3733	19	0.165	42.11994	57	0.883	150.55
3G029	0.159	21.41106	20	0.077	18.51994	58	0.558	88.45
3G032	0.097	11.8016	21	0.062	13.35264	61	0.3	44.87
3G038	0.12	15.29308	22	0.082	20.10946	65	0.517	81.21
3G039	0.219	31.24398	23	0.06	12.60274	69	0.126	18.1
3G042	0.17	23.17738	25	0.067	15.15524	C1	0.093	13.27
3G043	0.225	32.253	26	0.065	14.44558	C21	0.156	22.56
3G048	0.136	17.77466	27	0.215	53.43762	D2	0.274	40.74
3G055	0.147	19.50398	28	0.183	46.2981	D3	0.08	11.38
3G060	0.101	12.40196	29	0.297	70.58664	D5	0.289	43.12
3G063	0.107	13.30806	30	0.234	57.5369	D6	0.296	44.23
3G065	0.154	20.6139	31	0.159	40.6957	D8	0.356	53.93
3G077	0.12	15.29308	32	0.143	36.80764	D9	0.11	15.75
3G082	0.159	21.41106	33	0.061	12.97998	D10	0.278	41.37
3G084	0.136	17.77466	34	0.122	31.46322	D12	0.101	14.43
3G088	0.109	13.61154	35	0.171	43.5277	D13	0.715	90.41
3G089	0.157	21.09176	36	0.372	85.38002	D14	0.39	55.4
3G090	0.129	16.68392	37	0.13	33.5361	D16	0.1625	26.79
3G091	0.188	26.10378	38	0.045	6.10424	D17	0.4445	61.44
3G099	0.111	13.91572	39	0.121	31.20054	D18	0.4525	62.31
3G103	0.209	29.57234	40	0.0855	21.19224	D20	0.399	56.41
3G116	0.183	25.28648	41	0.1095	28.11564	D21	0.3905	55.46
3G117	0.18	24.79772	42	0.1015	25.89074	D22	0.3465	50.46
3G118	0.202	28.40978	43	0.1355	34.9339	D24	0.4835	65.68
3G120	0.185	25.613	44	0.0465	6.86116	D26	0.2065	33.11
3G123	0.346	53.51852	45	0.1295	33.40798	D28	0.853	105.37

3G124	0.114	14.3733	46	0.2	50.1317	D30	0.2705	41.41
3G126	0.158	21.25134	47	0.3775	86.44252	D31	0.502	67.68
3G127	0.126	16.21884	48	0.077	18.51994	D32	0.306	45.72
3G130	0.082	9.5781	49	0.2085	52.01318	E2	0.2785	42.4
3G138	0.088	10.46204	50	0.1155	29.74042	E4	0.442	61.16
3G140	0.107	13.30806	51	0.179	45.38098	E5	0.407	57.3
3G143	0.1075	13.38386	52	0.213	53.0006	E6	0.291	43.92
3G149	0.1045	12.92972	53	0.18	45.61084	E9	1.056	128.55
3G153	0.2045	28.82426	54	0.1855	46.86822	E10	0.2595	40.04
3G161	0.0845	9.9455	55	0.11	28.2524	E12	0.446	61.6
3G163	0.2965	44.61296	56	0.1435	37.37532	E13	0.355	51.44
3G168	0.082	9.5781	57	0.3175	74.69482	E16	0.333	48.9
3G169	0.1665	22.61352	58	0.127	32.76454	E18	0.3315	48.72
3G171	0.141	18.55844	59	0.1475	37.9154	E19	0.4505	62.09
3G175	0.2625	38.65952	60	0.075	17.86842	E20	0.285	43.19
3G180	0.092	11.05542	61	0.069	15.85132	E21	0.414	58.08
3G185	0.1	12.2516	62	0.048	7.58356	E23	0.233	36.65
3G186	0.1505	20.05804				E24	0.165	27.17
3G188	0.248	36.16212				E25	0.4965	67.08
3G206	0.0975	11.87648				E26	0.529	70.58
3G208	0.123	25.07696				E28	0.385	54.84
3G210	0.1	19.62134				E31	0.286	43.31
3G213	0.34	79.88254				E33	0.337	49.36
3G217	0.167	35.75628				E34	0.339	49.6
3G219	0.166	35.5105				E36	0.541	71.86
3G227	0.206	45.43876				E37	0.678	86.46
3G228	0.172	36.98718				E1	0.238	37.3
3G238	0.19	41.44442				E11	0.199	32.08
3G240	0.182	39.4585				E12	0.152	25.17
3G243	0.31	72.03416				E14	0.267	40.98
3G247	0.192	41.9421				E15	0.351	50.98
3G248	0.15	31.5964				E16	0.318	47.14
3G249	0.149	31.35296				E18	0.386	54.95
3G250	0.177	38.22126				E19	0.271	41.47
3G251	0.177	38.22126				E21	0.443	61.27
3G253	0.128	26.2755				E22	0.199	32.08
3G261	0.19	41.44442				E24	0.245	38.2
3G268	0.186	40.4505				E27	0.088	13.41
3G276	0.128	26.2755				E28	0.226	35.73
3G282	0.187	40.6988				E30	0.109	17.77
3G283	0.121	24.59872				E34	0.202	32.5
3G286	0.188	40.94722				E35	0.299	44.88
3G287	0.162	34.5287				E37	0.274	41.84
3G292	0.205	45.18826				E38	0.238	37.3

3G294	0.279	64.00212
3G300	0.149	31.35296
3G301	0.322	75.16488
3G312	0.131	26.99662
3G314	0.121	24.59872
3G317	0.245	55.2911
3G320	0.204	44.93788
3G326	0.106	21.03486
3G327	0.139	28.92664
3G342	0.061	10.63852
3G363	0.151	31.83998
3G367	0.199	43.68764
3G370	0.085	16.12106
3G375	0.136	28.2017
3G378	0.12	24.35986
3G382	0.148	31.10966
3G384	0.161	34.28358
3G385	0.157	33.30448
3G386	0.091	17.51514
3G390	0.104	20.56288
3G397	0.146	30.6235
3G398	0.213	47.19534
3G402	0.197	43.18832
3G408	0.1165	23.52526
3G416	0.1135	22.81162
3G417	0.103	20.3272
3G427	0.0825	15.5427
3G426	0.1795	38.8395
3G428	0.1015	19.97404
3G434	0.2065	45.56406
3G436	0.184	39.95426
3G437	0.139	28.92664
3G440	0.183	39.70632
3G441	0.1765	38.09772
3G453	0.144	30.13794
3G454	0.21	46.44186
3G457	0.3115	72.42486
3G460	0.0935	18.09842
3G463	0.197	43.18832
3G467	0.185	40.20232
3G468	0.11	29.52156
3G470	0.146	40.13704
3G481	0.151	41.58904
3G482	0.052	11.36266

E39	0.084	12.48
cancer hospital of shantou university medical collgee		
55	0.161	44.22
57	0.231	64.36
59	0.223	62.11
60	0.286	79.7
61	0.159	43.9
62	0.103	27.63
63	0.111	29.82
64	0.072	17.85
65	0.237	66.04
68	0.112	30.03
69	0.086	22.23
70	0.075	18.8
71	0.163	47.84
74	0.133	36.34
76	0.146	40.14
77	0.132	36.05
78	0.063	14.97
81	0.054	12.03
82	0.157	43.33
83	0.146	38.87
86	0.225	60.52
87	0.198	55.05
88	0.151	41.59
90	0.1505	41.44
91	0.0675	16.42
93	0.3225	89.81
94	0.2935	81.78
95	0.068	16.58
97	0.231	64.36
99	0.156	43.04
102	0.101	26.24
103	0.0992	22.23
105	0.2405	67.02
106	0.203	56.47
109	0.1985	55.19
111	0.182	50.5
112	0.22	43.39
115	0.2235	44.13
117	0.2005	39.19
118	0.2995	60.29
123	0.184	36.19

3G484	0.099	26.20582	126	0.241	47.87
3G490	0.066	15.94066	135	0.2845	57.12
3G493	0.117	31.61146	137	0.215	42.31
3G498	0.127	34.57356	144	0.263	53.72
3G502	0.156	43.03658	145	0.182	35.19
3G503	0.093	24.37868	147	0.2752	48.56
3G506	0.125	33.9832	150	0.149	32.72
3G507	0.089	23.15238	154	0.155	33.24
3G512	0.125	33.9832	158	0.073	19.04
3G521	0.094	24.6842	159	0.499	266.06
3G522	0.125	33.9832	179	0.214	44.27
3G528	0.129	35.16296	193	0.233	47.31
3G543	0.049	10.35444	216	0.129	24.54
3G544	0.14	38.38838	221	0.2001	40
3G552	0.109	29.22178	235	0.1335	24.74
3G555	0.13	35.4573	245	0.309	62.94
3G564	0.192	53.34854	266	0.249	50.33
3G570	0.217	60.42018	273	0.2985	59.92
			290	0.195	37.33
			295	0.401	134.75
			328	0.211	41.13
			386	0.439	195.54

Table S2. Association of L1CAM level with GC patients' clinicopathologic features.

Variables		L1CAM protein		
	**All cases**	**Low level**	**High level**	***P***
Age				
>58	82	43	39	0.137
≤58	66	26	40	
Gender				
Male	99	54	45	0.008
Female	49	15	34	
Smoke				
Yes	66	34	32	0.322
No	82	35	47	
Alcohol				
Yes	27	20	7	0.002
No	121	49	72	
Size of tumor				
>5cm	41	19	22	0.931
≤5cm	60	29	31	
Unknown	47	21	26	
Depth of tumor invasion				
T1+T2	19	12	7	0.023
T3+T4	122	51	71	
Unknown	7	6	1	
Lymph node metastasis				
N0	43	29	14	0.002
N1	25	12	13	
N2	41	16	25	
N3	31	7	24	
Unknown	8	5	3	
Distant metastasis				
Yes	19	8	11	0.99
No	123	58	65	
Unknown	6	3	3	
TNM stage				
Early stage (I+II)	46	30	16	0.006
Advanced stage (III+IV)	99	37	62	
Unknown	3	2	1	

Comparisons between groups were conducted with the use of person's Chi² tests. GC: gastric cancer.

Table S3. Association of L1CAM level with EJA patients' clinicopathologic features.

Variables	L1CAM protein			
	**All cases**	**Low level**	**High level**	***P***
Age				
>63	44	18	26	0.767
≤63	15	7	8	
Gender				
Male	45	20	25	0.758
Female	14	5	9	
Smoke				
Yes	13	6	7	0.750
No	42	18	24	
Unknown	4	1	3	
Diabetes				
Yes	3	2	1	0.543
No	52	22	30	
Unknown	4	1	3	
Adjuvant therapy				
Yes	23	10	13	0.578
No	25	9	16	
Unknown	11	6	5	
Depth of tumor invasion				
Tis	2	0	2	0.545
T1+T2	5	3	2	
T3+T4	42	18	24	
Unknown	10	4	6	
Lymph node metastasis				
N0	14	10	4	0.95
N1	8	5	3	
N2	7	5	2	
N3	20	12	8	
Unknown	10	7	3	
Distant metastasis				
Yes	14	7	7	0.804
No	35	14	21	
Unknown	10	4	6	
TNM stage				
Early stage (0+I+II)	13	7	6	0.637
Advanced stage (III+IV)	36	14	22	
Unknown	10	4	6	

Comparisons between groups were conducted with the use of person's Chi² tests. EJA: esophagogastric junction adenocarcinomas.
